# A pharmacokinetics‐based approach to the monitoring of patient adherence to atorvastatin therapy

**DOI:** 10.1002/prp2.856

**Published:** 2021-09-03

**Authors:** Gellért Balázs Karvaly, István Karádi, István Vincze, Michael N. Neely, Eszter Trojnár, Zoltán Prohászka, Éva Imreh, Barna Vásárhelyi, András Zsáry

**Affiliations:** ^1^ Laboratory of Mass Spectrometry and Separation Technology Department of Laboratory Medicine Semmelweis University Budapest Hungary; ^2^ Department of Internal Medicine and Hematology Semmelweis University Budapest Hungary; ^3^ Laboratory of Applied Pharmacokinetics and Bioinformatics The Saban Research Institute Keck School of Medicine University of Southern California Los Angeles California USA; ^4^ Buda Central Laboratory Department of Laboratory Medicine Semmelweis University Budapest Hungary; ^5^ Department of Laboratory Medicine Semmelweis University Budapest Hungary

**Keywords:** adherence, atorvastatin, metabolism, nonparametric pharmacokinetic model, pharmacokinetics, therapeutic drug monitoring

## Abstract

The inadequate adherence of patients whose hyperlipidemia is treated with atorvastatin (ATR) to medical instructions presents a serious health risk. Our aim was to develop a flexible approach based on therapeutic drug monitoring (TDM), nonparametric population pharmacokinetic modeling, and Monte Carlo simulation to differentiate adherent patients from partially and nonadherent individuals in a nonrandomized, unicentric, observational study. Sixty‐five subjects were enrolled. Nonparametric, mixed‐effect population pharmacokinetic models of the sums of atorvastatin and atorvastatin lactone concentrations (ATR+ATRL) and of the concentrations of the acid and lactone forms of ATR and its 2‐ and 4‐hydroxylated pharmacologically active metabolites (ATR+MET) were elaborated by including the TDM results obtained in 128 samples collected from thirty‐nine subjects. Monte Carlo simulation was performed based on the elaborated models to establish the probabilities of attaining a specific ATR+ATRL or ATR+MET concentration in the range of 0.002–10 nmol (mg dose)^−1^ L^−1^ at 1–24 h postdose by adherent, partially adherent, and nonadherent patients. The results of the simulations were processed to allow the estimation of the adherence of further 26 subjects who were phlebotomized at sampling times of 2–20 h postdose by calculating the probabilities of attaining the ATR+ATRL and ATR+MET concentrations measured in these subjects in adherent, partially adherent, and nonadherent individuals. The best predictive values of the estimates of adherence could be obtained with sampling at early sampling times. 61.54% and 38.46% of subjects in the adherence testing set were estimated to be fully and partially adherent, respectively, while in all cases the probability of nonadherence was extremely low. The evaluation of patient adherence to ATR therapy based on pharmacokinetic modeling and Monte Carlo simulation has important advantages over the collection of trough samples and the use of therapeutic ranges.

## Graphical abstract

We set forth a pharmacokinetics‐based clinical laboratory procedure and decision‐making algorithm for the therapeutic monitoring of atorvastatin concentrations at any time that has passed from drug intake to sample collection.

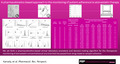

**Abbreviations**
2OATR: 2‐hydroxyatorvastatin
2OATRL: 2‐hydroxyatorvastatin lactone
4OATR: 4‐hydroxyatorvastatin
4OATRL: 4‐hydroxyatorvastatin lactone
anti‐HMGCR: anti‐3‐hydroxy‐3‐methylglutaryl‐coenzyme A reductase antibody
ATR: atorvastatin
ATRL: atorvastatin lactone
AUC: area under the receiver‐operating characteristic curve
c_ini_
: initial concentration
CK: creatine kinase
F: oral bioavailability
FPR: false positive rate
FST: first sampling time
GOT: glutamate‐oxaloacetate transaminase
GPT: glutamate‐pyruvate transaminase
HDL‐C: high‐density lipoprotein cholesterol
HMG‐CoA‐R: 3‐hydroxy‐3‐methylglutaryl‐coenzyme A reductase
K_a_
: absorption rate constant
K_e_
: elimination rate constant
LDH: lactate dehydrogenase
LDL‐C: low‐density lipoprotein cholesterol
ROC: receiver‐operating characteristic curve
TDM: therapeutic drug monitoring
TG: triglyceride
TPR: true positive rate
V: volume of distribution
V/F: apparent volume of distribution

## 1 INTRODUCTION

Cardiovascular events remain the leading cause of death among adults, and their occurrence is strongly associated with dyslipidemia.^1^, ^2^ As a core component of their prevention, individuals who have abnormal cholesterol levels should be maintained on maximally tolerable statin therapy.^3^ Statins are potent competitive inhibitors of 3‐hydroxy‐3‐methylglutaryl‐coenzyme A reductase (HMG‐CoA‐R). A recent meta‐analysis has shown that, in patients who had pathological serum lipid levels, statin‐based therapy resulted in a 24% reduction in the occurrence of major coronary events for each 1.0 mmol/L decrease in low‐density lipoprotein cholesterol (LDL‐C) levels, regardless of age.^3^, ^4^, ^5^

**FIGURE 1:**
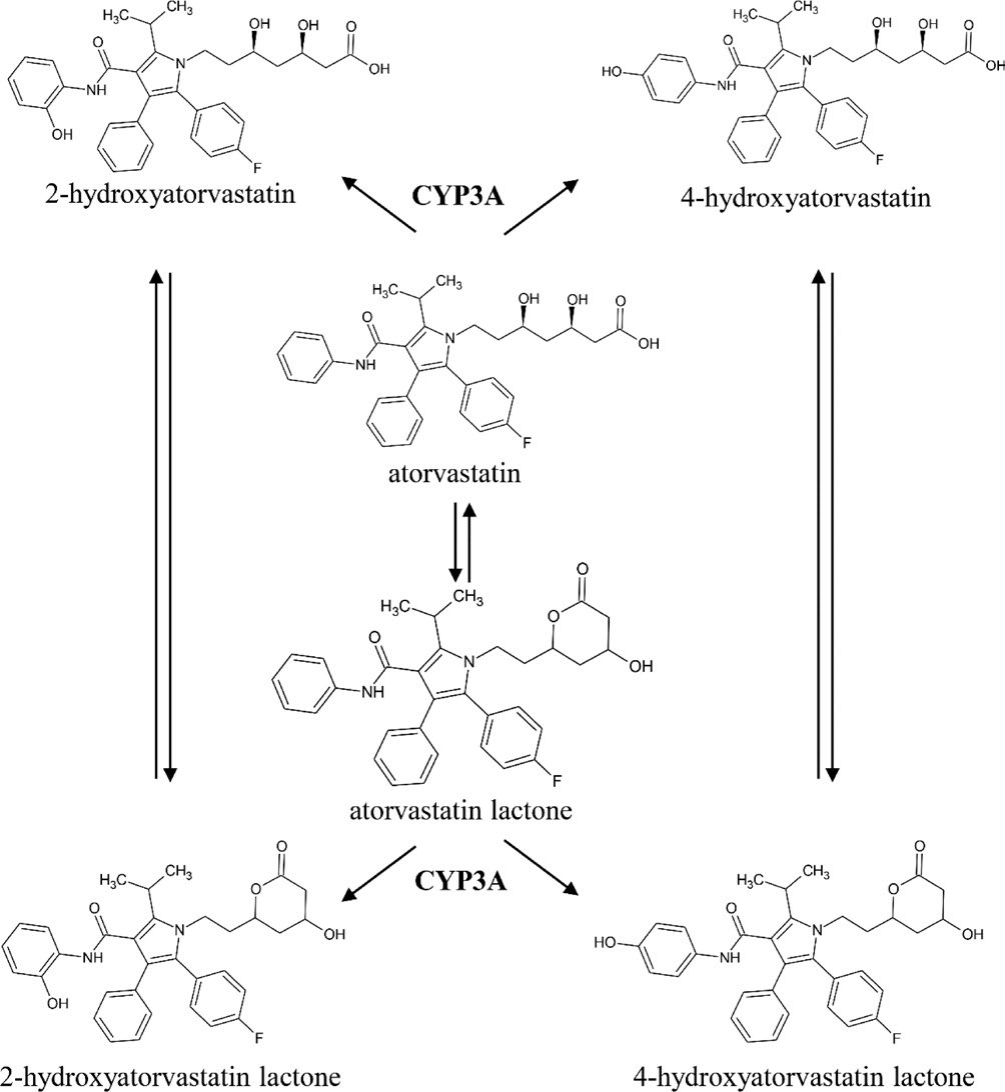
Overview of atorvastatin metabolism. CYP3A, Cytochrome P 450 3A

The primary advantage of administering atorvastatin {(3*R*,5*R*)‐7‐[2‐(4‐fluorophenyl)‐3‐phenyl‐4‐(phenylcarbamoyl)‐5‐propan‐2‐ylpyrrol‐1‐yl]‐3,5‐dihydroxyheptanoic acid, ATR} over other statins is the negligible extent of its renal excretion which eliminates the need of dose adjustment due to renal impairment. However, the cytochrome 450 3A4‐mediated hepatic metabolism of ATR is extensive, resulting in the formation of 2‐hydroxy‐ (2OATR) and 4‐hydroxy atorvastatin (4OATR), as well as of the lactonized forms (ATRL, 2OATRL, and 4OATRL, Figure 1) which causes its pharmacokinetics to be complex.^6^, ^7^, ^8^, ^9^, ^10^

According to the American Heart Association, the incidence of statin‐associated muscular symptoms and statin‐induced diabetes mellitus, conditions prompting the discontinuation of statin therapy, is approximately <0.1% and 0.2% per year of therapy, respectively.^11^ However, a considerably larger proportion of patients discontinues ATR therapy beyond the first year of treatment, presenting a very serious health risk on the long term.^12^, ^13^, ^14^ The underlying reasons for abandoning the otherwise safe and comfortable measures are currently unknown. Consequently, establishing an evidence‐based procedure for the verification of adherence would be an important first step in identifying these reasons and, ultimately, in improving the clinical outcome of ATR therapy.

Therapeutic drug monitoring (TDM) is a tool for the clinical verification of patient adherence to medical instructions with relatively small ambiguity in its performance as compared to other approaches. Recently, a small‐scale study conducted with 24 participants has proposed a cut‐off concentration of 0.05 nmol (mg dose)^−1^ L^−1^ regarding the sum of ATR+ATRL concentrations assayed 24 h post dose to identify individuals who missed at least the last dose (referred to as “partial adherence”) with 100% sensitivity and 83% specificity, and those who missed the last three doses or more (referred to as “nonadherent patient”) with 100% sensitivity and 100% specificity. Due to the interindividual variability of metabolism, it was nevertheless proposed that the sums of ATR, ATRL, 2OATR, 2OATRL, 4OATR, and 4OATRL (ATR+MET) concentrations, taken as a single entity, should also be evaluated with a 0.1 nmol (mg dose)^−1^ L^−1^ cut‐off concentration indicating partial adherence.^15^ These cut‐off concentrations were not based on pharmacokinetic evaluation.

The uncertainties associated with accurate dose times in the outpatient setting confound comparison of a measured concentration to a range or threshold that depends on a specific elapsed time between dose and sample, especially if the target timing is inconvenient for the patient, for example, very early in the morning or late at night. In contrast, population pharmacokinetic modeling based on flexibly timed sampling, combined with Monte Carlo simulation to assess the probability of obtaining such a concentration, allows a more robust estimate than the assessment of trough or other specifically timed concentrations by transforming adherence into a continuous probability rather than a categorical variable. Earlier, Neely has used this approach successfully for verifying the adherence of a pediatric patient to her voriconazole dosing regimen.^16^ We set forth a pharmacokinetics‐based clinical laboratory procedure and decision‐making algorithm for the TDM of ATR at any time that has passed from drug intake to sample collection.

## 2 MATERIALS AND METHODS

### 2.1 Study design and demographics

**FIGURE 2:**
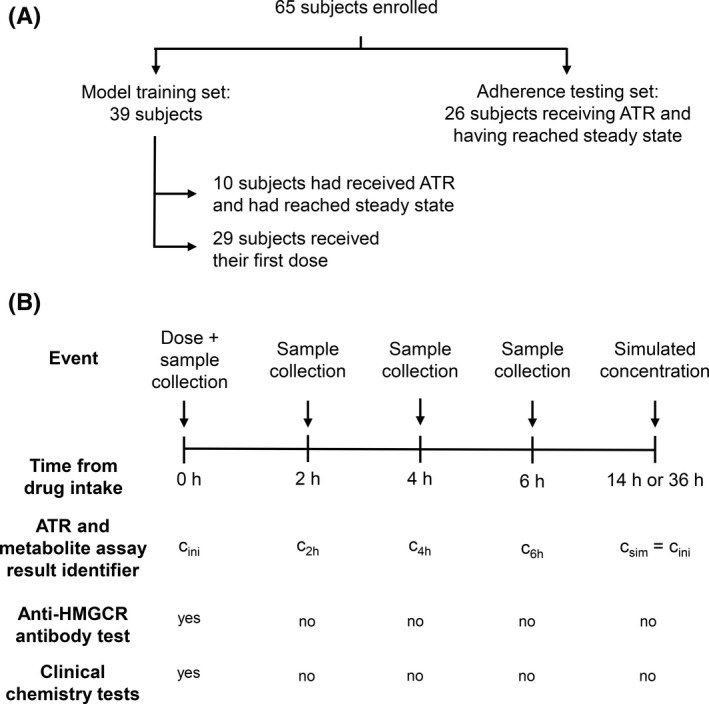
Scheme of the study design. (A) Structure of the study. (B) Dose administration and sampling scheme. ATR, atorvastatin. Anti‐HMGCR: anti‐3‐hydroxy‐3‐methylglutaryl‐coenzyme A reductase antibody. c_ini_: initial concentration of ATR+ATRL or ATR+MET. c_sim_: simulated concentration of ATR+ATRL or ATR+MET

This research was a nonrandomized, unicentric, observational clinical study approved by the Regional and Institutional Committee of Science and Research Ethics, Semmelweis University, Budapest, Hungary (197/2017, October 2, 2017). The study population comprised 65 individuals who were prescribed Atoris, a film‐coated tablet containing atorvastatin calcium salt and 5.405 mg lactose for each mg of ATR (KRKA, d.d, Novo mesto, Slovenia), at the Unit of Cardiology, Department of Internal Medicine and Hematology, Semmelweis University, with the primary surrogate end‐point of therapy being the change in LDL‐C concentrations (Figure 2). Demographic information on the recruited subjects is summarized in Table 1 (detailed information is provided in Supporting Information 1). Interaction with patients was made on a single occasion for the purposes of the study. Following physical examination and obtaining the written informed consent of the subjects, anthropometric data, habits related to drug intake, physical status, and posology were recorded.

**TABLE 1:** Patient demographics

	Pharmacokinetic model training set	Adherence testing set
Number of subjects	39	26
Age (years)	75 (29–88)	72.5 (47–82)
BMI (kg/m^2^)	28.4 (17.1–44.1)	not recorded
LDL cholesterol (mmol/L)	2.28 (1.24–4.50)	2.26 (1.35–3.52)
HDL cholesterol (mmol/L)	0.92 (0.58–1.72)	1.49 (0.77–2.01)
Triglycerides (mmol/L)	1.17 (0.43–24.3)	1.16 (0.50–6.14)
Total cholesterol (mmol/L)	3.50 (1.27–12.20)	4.10 (2.40–5.80)
Creatinine kinase (U/L)	96.5 (15.0–224.0)	89 (39–316)
GPT (U/L)	22 (12–121)	18.5 (8–33)
GOT (U/L)	26 (12–101)	20.5 (13–39)
LDH (U/L)	188 (88–356)	191 (82–255)
Serum creatinine (µmol/L)	102 (47–501)	73 (43–164)
Number of subjects receiving each dose of atorvastatin		
10 mg	0	1
20 mg	28	18
40 mg	11	5
80 mg	0	2

Median values are shown with ranges in parentheses.

BMI, body‐mass index; GOT, glutamate‐oxaloacetate transaminase; GPT, glutamate‐pyruvate transaminase; HDL, high‐density lipoprotein; LDH, lactate dehydrogenase; LDL, low‐density lipoprotein

Thirty‐nine subjects comprised the *model training set*. Twenty‐nine subjects took their first dose of ATR (n = 24 and n = 5 receiving 20 mg and 40 mg, respectively) under the supervision of the recruiting clinical team. Ten subjects had a history of treatment with ATR (n = 4 and n = 6 receiving 20 mg and 40 mg, respectively). These subjects were patients well known to the recruiting clinicians, and had been cooperative regarding their treatment. They had previously been requested to omit the last dose of ATR before the visit, were interrogated on the time of the last intake, and took their dose in front of the recruiting clinical team. A single subject disobeyed the instruction and took 20 mg ATR the evening before the visit.

Twenty‐six subjects, all of whom had been on chronic ATR therapy with 10‐mg, 20‐mg, 40‐mg, or 80‐mg doses (n = 1, n = 18, n = 5, and n = 2, respectively), comprised the *adherence testing set*.

### 2.2 Laboratory procedures

Laboratory assays were conducted at the Department of Laboratory Medicine, Semmelweis University. A blood sample without any additive was drawn into a 6‐mL collection tube immediately before drug intake (Figure 2, c_ini_). Three further blood samples, 6 ml each, were collected from subjects of the model training set at 2, 4, and 6 h post dose (c_2_, c_4_, and c_6_, respectively, Figure 2). Subjects in the adherence testing set donated a single 2‐mL native blood sample.

All samples were centrifuged at 4000 *g* for 10 min immediately following their collection. 200 µL serum separated from c_ini_, c_2_, c_4_, and c_6_ samples, as well as of those collected from subjects of the adherence testing set, was transferred to a 2‐mL cryo vial, was frozen, and was allocated for the analysis of ATR and its metabolites. Sera not analyzed immediately were frozen to −75°C until analysis. The concentrations of ATR, ATRL, 2OATRL, 2OATRL, 4OATR, and 4OATRL were evaluated using liquid chromatography‐tandem mass spectrometry as described elsewhere.^17^ For appropriate precision‐based weighting of concentrations in the modeling process, the equations to account for assay error as the standard deviation (S.D.) of measured values were established from assay validation data.^17^, ^18^ The equations S.D. =8.26 × 10^−5^ + 3.53 × 10^−2^ × concentration and S.D. = 3.86 × 10^−5^ + 3.32 × 10^−2^ × concentration were used for modeling ATR+ATRL and ATR+MET, respectively.

A 1‐mL aliquot of the c_ini_ sample was used to assess the concentration of the antibody forming against HMG‐CoA‐R (anti‐HMGCR). Briefly, 96‐well enzyme‐linked immunosorbent assay plates were coated with recombinant human HMG‐CoA‐R (Sigma‐Aldrich Kft., Budapest, Hungary). Following washing and the blocking of nonspecific binding sites, human serum was added. Horseradish peroxidase‐labeled rabbit anti‐human antibody (Dako, Glostrup, Denmark) was applied as the secondary antibody. Bound IgG was detected using 3,3′,5,5′‐tetramethylbenzidine as substrate at λ = 450 nm (reference at λ = 620 nm). The authors understand that, currently, there is sparse evidence that anti‐HMGCR concentrations are related to the adverse events associates with ATR therapy, which may also be the result of high ATR exposure.^19^

Another 1‐mL aliquot of the C_ini_ sample was employed for performing automated clinical chemistry parameters including creatine kinase (CK), glutamate‐oxaloacetate transaminase (GOT), glutamate‐pyruvate transaminase (GPT), high‐density lipoprotein cholesterol (HDL‐C), lactate dehydrogenase (LDH), LDL‐C, serum creatinine, total cholesterol and triglycerides (TG) on a Beckmann Coulter Clinical Chemistry Analyzer AU680, using laboratory reagents manufactured by Beckmann Coulter (purchased from Beckman Coulter Magyarország Kft.).

### 2.3 Pharmacokinetic modeling

Nonparametric, nonlinear mixed‐effect single‐compartment population pharmacokinetic models were constructed following the calculation of (1) the sum of measured atorvastatin and atorvastatin lactone concentrations (ATR+ATRL), and (2) the sum of the measured concentrations of atorvastatin, atorvastatin lactone, 2‐hydroxyatorvastatin, 2‐hydroxyatorvastatin lactone, 4‐hydroxyatorvastatin, and 4‐hydroxyatorvastatin lactone (ATR+MET) in 128 samples of the 39 subjects in the model training set, and by applying the nonparametric adaptive grid algorithm included in the software package Pmetrics^TM^ running in the R environment, written by one of the authors (MNN).^20^, ^21^, ^22^ Absorption rate coefficient (k_a_), volume of distribution (V,) and elimination rate coefficient (k_e_) were included as random effects. Absolute oral bioavailability (F) was included as a fixed effect (F = 0.125). The apparent volume of distribution was calculated as V/F, equal to V/0.125. The modeled parameter ranges were established as part of model optimization. Pre‐dose concentrations (c_ini_) measured in the 10 subjects who had received earlier doses of ATR were used to set the initial conditions (#ini) for the pharmacokinetic model and were also assumed to be equal to the concentration simulated at the end of the dosing interval [36 h, except for a single subject (14 h)] after the witnessed dose (Figure 2, Supporting information 2). Due to the required syntax of input model files created for population pharmacokinetic modeling with Pmetrics^TM^, c_ini_ had to be set as a technical covariate in the model file, but was not considered or tested as a true pharmacokinetic model covariate. The input data files are provided as Supporting Information 3 and 4.

The linear correlation of candidate continuous covariates (age, body‐mass index, serum creatinine, serum total cholesterol, CK, LDL‐C, TG, HDL‐C, LDH, GOT, and GPT anti‐HMGCR) versus the individual posterior values of random effects obtained in the final pharmacokinetic models was tested. The threshold for considering a candidate covariate for further evaluation was r = 0.7. The relationship between the random effects and ATR dose or gender was explored by performing Wilcoxon's rank sum test with non‐exact *p*, with *p* = .010 considered as the threshold for further evaluation as a categorical covariate. Additional noise related to model misspecification or other process noise was estimated by applying an additive error model where λ (set to 0.01 in both models) represents the clinical and preanalytical sources of error, while C_0_ and C_1_ are coefficients of the assay error equation.^23^ The final weighting for each concentration was 1/(λ + C0 + C1 × concentration). Two arguments of the “NPrun()” function were set to non‐default values: the convergence criterion (“ode”) was 0.0001, and the allowed number of iterations (“cycles”) was 5000.

### 2.4 Monte Carlo simulations

Based on the outputs of the final pharmacokinetic models, Monte Carlo simulations were conducted using Pmetrics^TM^. The number of simulated patients was 50000 (10000 per ATR dose), with simulated doses of 5 mg (8.95 µmol), 10 mg (17.9 µmol), 20 mg (35.8 µmol), 40 mg (71.6 µmol), and 80 mg (151.2 µmol). The administration of 14 doses of ATR, once daily, was simulated before collecting the first sample at the first sampling time (FST). FSTs were simulated between 1 and 24 h in steps of 1 h, with further sampling times set at FST+24 h to simulate the omission of the last dose (corresponding to partial adherence), and at FST+72 h to simulate the omission of the last three doses (representing nonadherence).

### 2.5 Data evaluation

Microsoft Excel 2013 and R were used for data evaluation. The results of anti‐HMGCR measurements were calculated using GraphPad Prism 6.00 (GraphPad Software). Clinical chemistry assay results were calculated by the software of the chemical analyzers.

Visual predictive checks and functions integrated into Pmetrics^TM^ and the “stats” package of R were employed for the evaluation and optimization of the constructed population pharmacokinetic models. Linear regression was performed on observed versus predicted concentrations. The mean weighted squared prediction error obtained for alternative models was compared. Student's *t* test was conducted to display the statistical difference of the mean weighted prediction error (bias) from zero. Kruskal–Wallis test was employed for establishing the deviation of the residuals from normal distribution. The distribution of the probabilities of random effects was checked visually with an accepted shrinkage range of 0%–30%.^24^ The comparison was based on the number of support points, −2 × log likelihoods, the Akaike and the Bayesian information criteria. The models were considered to be statistically different from each other at *p* < .05. Linear correlation between the individual posterior values of random effects and quantitative demographic data [age and body‐mass index], laboratory assay results (serum creatinine, total cholesterol, CK, LDL‐C, TG, HDL‐C, LDH, GOT, and GPT) and anti‐HMGCR was investigated. The relationship with dose (20 or 40 mg) and gender was tested by performing unpaired Wilcoxon tests with non‐exact p‐values.

Dose‐normalized simulated concentrations were employed for generating receiver‐operating characteristic (ROC) curves of ATR+ATRL and ATR+MET to discriminate between full adherence and partial adherence, as well as between partial adherence and nonadherence using Microsoft Excel 2016 and an R script written by one of the authors (GBK). The true positive (TPR, corresponding to sensitivity) and false positive rates (FPR, corresponding to 1‐specificity) of ATR+ATRL and of ATR+MET were established by using Equations (1) and (2) for each cut‐off concentration between 0.002 and 10 nmol (mg dose)^−1^ L^−1^, in steps of 0.002 nmol (mg dose)^−1^ L^−1^.

(1) $$\mathrm{TPR}\;=\;\frac{\mathrm{number}\;\mathrm{of}\;\mathrm{subjects}\;\mathrm{with}\;\mathrm{dose}‐\mathrm{normalized}\;\mathrm{concentrations}\;\mathrm{lower}\;\;\mathrm{than}\;\mathrm{the}\;\mathrm{cut}‐\mathrm{off}\;\mathrm{in}\;\mathrm{the}\;\mathrm{less}\;\mathrm{adherent}\;\mathrm{group}}{50000}$$

(2) $$\mathrm{FPR}\;=\;\frac{\mathrm{number}\;\mathrm{of}\;\mathrm{subjects}\;\mathrm{with}\;\mathrm{dose}‐\mathrm{normalized}\;\mathrm{concentrations}\;\mathrm{lower}\;\;\mathrm{than}\;\mathrm{the}\;\mathrm{cut}‐\mathrm{off}\;\mathrm{in}\;\mathrm{the}\;\mathrm{more}\;\mathrm{adherent}\;\mathrm{group}}{50000}$$

ROC curves were created by plotting TPR versus FPR for each sampling protocol. The areas under the ROC curves (AUCs) were calculated using the trapezoid method. The recommended cut‐off concentrations of ATR+ATRL and ATR+MET at a given sampling time are the ones for which the difference between sensitivity and specificity was the smallest (termed as “optimal difference”) [Equation (3)].

(3) $$\mathrm{optimal}\;\mathrm{difference}\;=\;\min(|{\mathrm{specificity}}_{i}‐{\mathrm{sensitivity}}_{i}|)$$

where i is the ith cut‐off concentration [i =1 for 0.002 nmol (mg dose)^−1^ L^−1^, and i = 5000 for 10 nmol (mg dose)^−1^ L^−1^), Δi = i(n)‐i(n‐1) =0.002].

## 3 RESULTS

### 3.1 Pharmacokinetic modeling

**FIGURE 3:**
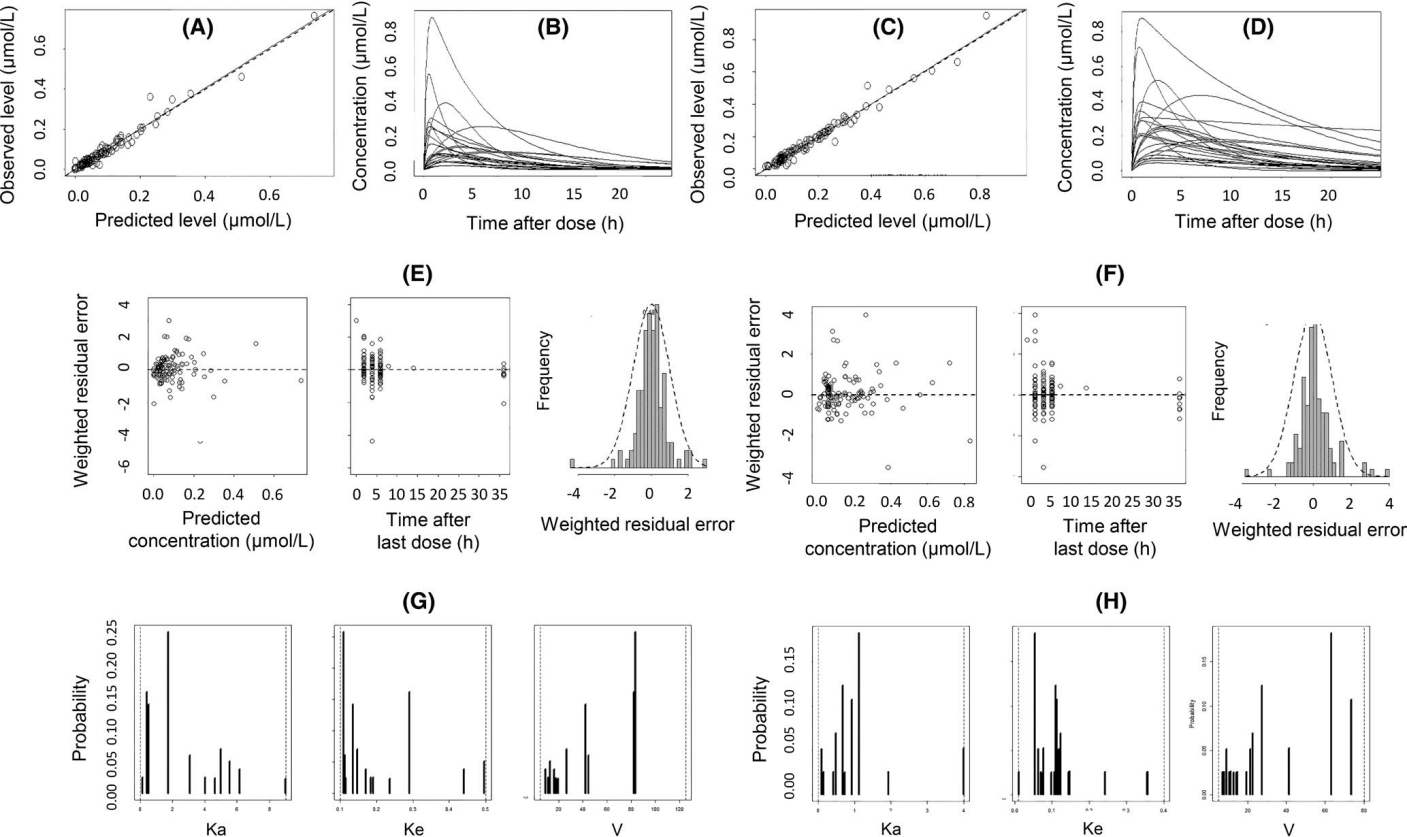
Characteristics of the single‐compartment population pharmacokinetic models of atorvastatin+atorvastatin lactone, and of all assayed atorvastatin‐related entities (ATR+MET). (A) Relationship between the predicted and observed sums of atorvastatin+atorvastatin lactone concentrations (ATR+ATRL, r = 0.981). (B) Predicted 24‐h ATR+ATRL concentration curves in the subjects included in the model training set. (C) Relationship between the predicted and observed sums of the concentrations of all atorvastatin‐related entities (r = 0.985). (D) Predicted 24‐h concentration curves of ATR+MET in the subjects included in the model training set. (E) Residual plots of ATR+ATRL. (F) Residual plots of ATR+MET. (G) Bar charts showing the support point components of the population pharmacokinetic model of ATR+ATRL. (H) Bar charts showing the support point components of the population pharmacokinetic model of ATR+MET. Ka, absorption rate constant. Ke, elimination rate constant. V, volume of distribution

The main characteristics of the constructed population pharmacokinetic models are displayed in Figure 3 and Table 2. Further details are provided in Supporting Information 5. Concerning ATR+ATRL, significant correlation was identified between individual posterior K_a_ values and CK (*p* = .22, r = 0.366), as well as between V/F and gender (*p* = .020), serum total cholesterol (*p* = .024, r = 0.361), LDL‐C (*p* = .032, r = 0.343), and HDL‐C (*p* = .025, r = 0.358). Concerning ATR+MET, the correlation of individual posterior K_e_ values with dose (*p* = .026), as well as of V/F with age (*p* = .019, r = 0.374), serum total cholesterol (*p* = .021, r = 0.370), and LDL‐C (*p* = .029, r = 0.350) was significant. Nevertheless, the correlation was weak in all cases (r ≤ 0.374), therefore, only the technical covariate c_ini_ was included in the final population pharmacokinetic models (Supporting Information 6).^25^

**TABLE 2:** Output characteristics of the built mixed‐effect population pharmacokinetic models

	ATR+ATRL	ATR+MET
Slope of the regression line of predicted versus observed concentrations (95% confidence interval)	1.01 (0.98–1.05)	1.01 (0.98–1.05)
Intercept of the regression line of predicted versus observed concentrations (95% confidence interval)	−0.001 (−0.006–0.003)	−0.003 (−0.009–0.003)
Correlation coefficient of predicted versus observed concentrations	0.981	0.985
Median K_a_ (range, 1/h)	1.74 (0.131–9.00)	1.12 (0.048–4.00)
Median K_e_ (range, 1/h)	0.135 (0.108–0.496)	0.110 (0.063–0.357)
Median V/F (range, L)	358 (73.7–668)	220 (57.3–589)

F, bioavailability; K_a_, absorption rate constant; K_e_, elimination rate constant; V/F, apparent volume of distribution following oral administration.

### 3.2 Monte Carlo simulations

**FIGURE 4:**
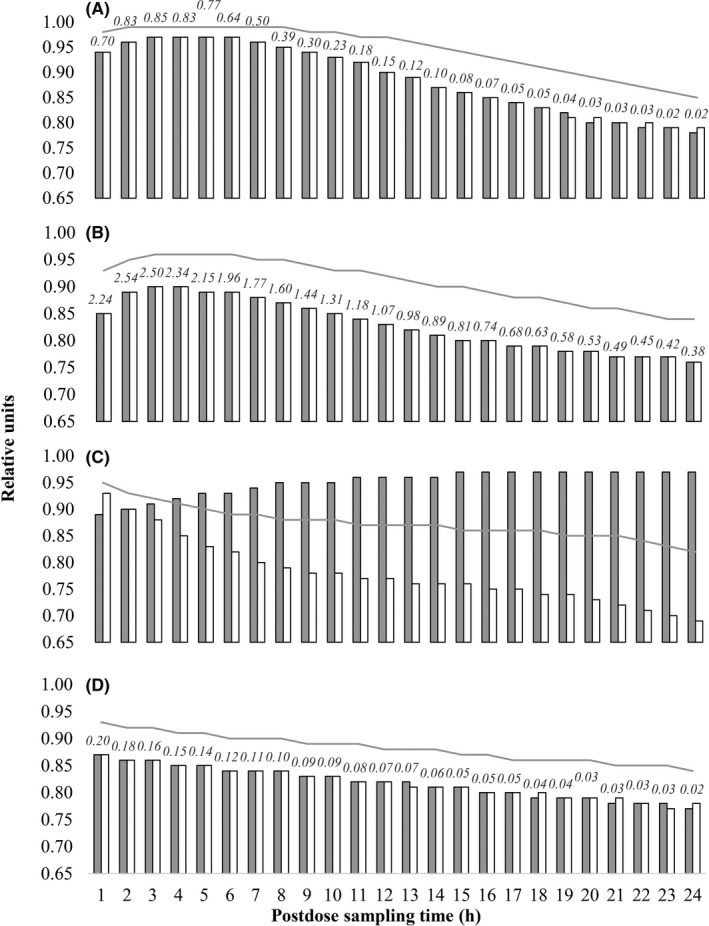
Performance of adherence estimation based on the results of Monte Carlo simulations in a 24 h sampling time period following the intake of the last dose of ATR. A 2‐week history of treatment prior to sampling was simulated. Gray and white bars represent the sensitivity and specificity calculated at the cut‐off concentrations (Equation 3). The gray lines show the trends of the areas under the receiver‐operating characteristic curve obtained at each sampling time. The cut‐off concentrations where the minimal differences of sensitivity and specificity were calculated (Equation 3) are shown by numbers in italic. The performance of the calculations to discriminate between full and partial adherence, as well as between partial adherence and nonadherence, respectively, are shown for ATR+ATRL (A, C) and for ATR+MET (B, D). In (C), all data were obtained at a cut‐off concentration of 0.002 nmol (mg dose^−1^) L^−1^

Cut‐off concentrations of ATR+ATRL and of ATR+MET where the optimal differences were obtained, along with the respective sensitivities, specificities, and the areas under the receiver‐operating characteristic curves calculated at these cut‐off values, are displayed with a 1‐hour resolution concerning sampling times of 1–24 h postdose (Figure 4). The largest AUCs of ATR+ATRL were obtained for the discrimination between full and partial adherence at postdose sampling times of 2–8 h. In this sampling time range, the optimal cut‐off concentrations were 0.386–0.850 nmol (mg dose)^−1^ L^−1^. The largest AUC of ATR+MET was obtained at 3–6 h with optimal cut‐off concentrations of 1.958–2.496 nmol (mg dose)^−1^ L^−1^. Concerning the discrimination between partial adherence and nonadherence, the largest AUCs were obtained at 1 h both for ATR+ATRL and for ATR+MET, followed by a monotonous decline. The optimal cut‐off concentrations of ATR+ATRL were the smallest simulated value, 0.002 nmol (mg dose)^−1^ L^−1^, irrespective of the sampling time. The optimal ATR+MET cut‐off concentration was 0.200 nmol (mg dose)^−1^ L^−1^ at 1 h, followed by a monotonous decline to 0.022 nmol (mg dose)^−1^ L^−1^ by 24 h.

### 3.3 Evaluation of the adherence of individual subjects

The probabilities of full, partial, and nonadherence calculated for the subjects of the adherence testing set are displayed in Table 3. Three subjects reported taking ATR on the morning of visit. Twenty‐three subjects reported to have ingested their last dose the day before. Based on individual ATR+ATRL and ATR+MET concentrations which were compared the results of the Monte Carlo simulations, partial adherence was suspected in 10 subjects (38.5%) of the adherence testing set. The individual concentrations provided no ground for assuming nonadherence for any of the subjects (Table 3).

**TABLE 3:** Probabilities of attaining the measured ATR+ATRL, as well as the total ATR+MET concentrations with various degrees of adherence (full adherence, missing last dose: partial adherence, or missing at least three doses: nonadherence)

Subject identifier	Dose (mg)	Time to sampling (h)	Assayed concentrations normalized to the dose prescribed [nmol (mg dose)^−1^ L^−1^]		% probability of attaining the measured concentrations with various degrees of adherence						Classification
					ATR+ATRL			ATR+MET			
			ATR+ATRL	ATR+MET	Fully adherent	Partially adherent	Nonadherent	Fully adherent	Partially adherent	Nonadherent	
A1	40	12	0.312	0.775	79.74%	3.62%	0.03%	95.29%	25.67%	2.65%	PA
A2	20	16	0.312	0.782	71.59%	2.31%	0.30%	78.48%	19.04%	2.44%	A
A3	20	12	0.064	0.174	97.69%	29.54%	1.13%	99.98%	64.82%	9.87%	PA
A4	20	16	1.032	2.474	13.59%	0.33%	0.00%	33.69%	3.45%	1.03%	A
A5	40	16	0.015	0.117	96.84%	59.72%	1.79%	97.79%	63.84%	11.57%	PA
A6	20	15	0.497	1.081	61.03%	1.47%	0.20%	75.18%	13.75%	1.97%	A
A7	20	16	0.492	1.393	55.38%	1.33%	0.20%	70.14%	8.79%	1.60%	A
A8	20	15	0.323	1.253	73.45%	2.49%	0.30%	73.84%	11.15%	1.74%	A
A9	20	16	0.145	0.538	78.40%	5.63%	0.61%	89.94%	28.15%	3.36%	PA
A10	20	16	0.378	0.960	66.44%	1.86%	0.28%	75.36%	14.93%	2.08%	A
A11	20	18	0.222	0.926	72.19%	2.75%	0.42%	73.61%	13.56%	2.06%	A
A12	40	2	0.274	0.572	100%	21.8%	0.56%	100%	63.19%	4.87%	PA
A13	20	13	0.533	1.098	68.72%	1.63%	0.19%	78.00%	15.48%	2.00%	A
A14	40	12	0.223	0.367	83.57%	5.56%	0.49%	98.40%	45.95%	5.33%	PA
A15	80	20	0.567	0.952	24.33%	0.88%	0.12%	68.63%	11.55%	1.96%	A
A16	20	10	0.294	0.623	87.72%	5.15%	0.42%	98.98%	36.03%	3.49%	PA
A17	10	18	0.364	0.973	57.53%	1.61%	0.28%	72.97%	12.83%	2.00%	A
A18	40	12	0.533	2.176	72.33%	1.87%	0.20%	67.12%	5.81%	1.16%	A
A19	20	12	0.734	1.679	61.47%	1.21%	0.09%	74.19%	8.87%	1.44%	A
A20	20	14	0.227	0.670	78.57%	4.18%	0.46%	91.40%	25.96%	2.89%	PA
A21	20	12	0.154	0.398	89.12%	9.04%	0.65%	98.20%	43.84%	4.95%	PA
A22	20	12	0.617	1.722	68.86%	1.49%	0.15%	73.84%	8.49%	1.42%	A
A23	20	2	1.562	3.560	84.74%	1.26%	0.02%	73.48%	5.60%	0.95%	A
A24	20	2	0.949	2.351	94.51%	3.29%	0.10%	93.20%	12.61%	1.29%	PA
A25	80	15	0.850	1.778	35.80%	0.82%	0.06%	63.57%	6.53%	1.29%	A
A26	20	12	0.700	1.727	63.75%	1.26%	0.11%	73.82%	8.46%	1.41%	A

ATR+ATRL, atorvastatin and atorvastatin lactone; ATR+MET, atorvastatin and all assayed metabolites; A, fully adherent; PA, partially adherent (missed last dose).

## 4 DISCUSSION

The verification of patient adherence is a prerequisite for the efficient guidance of pharmacotherapy. Kristiansen and his coworkers have recently made an effort to discriminate between adherent, partially adherent, and nonadherent patients to whom ATR had been prescribed by assaying ATR and its hydroxylated metabolites.^15^ They found that the combined concentrations of ATR+ATRL and ATR+MET were more relevant than those of individual analytes. Clear‐cut cut‐off values were proposed with sampling at 24 h postdose. As a step forward, we recommend the pharmacokinetics‐based evaluation of ATR+ATRL and of ATR+MET in order to achieve full flexibility regarding the time of sampling and to enable probability‐based decisions on an individual basis—a concept central to precision pharmacotherapy. The offered flexibility is very important because the time passing between sampling and dose intake may be difficult to control. Planning the sampling time ahead may now be replaced efficiently with simply recording the times of drug intake and of sample collection, a process prone to less error. In addition, due to the trend of drug concentrations following the administration of an oral tablet, sampling at 24 h postdose is suboptimal for discriminating among the various levels of adherence in comparison to the application of earlier sampling times (Figure 4).

The presented pharmacokinetics‐based approach relies on the measurement of ATR and all five metabolites. In certain situations, the estimation of therapy adherence may be conducted by considering only ATR+ATRL concentrations. In certain cases the discrimination of partial adherence from nonadherence can be a dichotomous judgment based on the presence or absence of ATR+ATRL in the sample, especially if an early sampling time is employed (Figure 4). Nevertheless, the evaluation both ATR+ATRL and ATR+MET seems to be of benefit in many cases due to the slower turnover of the hydroxylated metabolites.

Patients who undergo adherence testing will, assumably, have taken multiple doses of ATR. To avoid the exclusion of potentially important sources of interindividual variability associated with prolonged ATR use, such as comedication or gut microbial differences, a mixed population of subjects who had a history of treatment, and who received the first dose during the visit, was formed. Importantly, this decision could be made as the Pmetrics^TM^ package could handle repeat dosing for population modeling and simulation. The decision to not limit the administered dose of ATR to a single quantity was based on its linear pharmacokinetics. ATR+ATRL and ATR+MET were handled as a single chemical entity for modeling. Two‐compartment models are often superior in describing the pharmacokinetics of lipophilic drugs such as ATR, but the employed sparse sampling scheme only allowed the elaboration of single‐compartment models, with the known risk of underestimating concentrations in the terminal phase. Both elaborated models were satisfactory in performance. The correlation between observed and predicted concentrations was strong, and the slopes and intercepts of the regression lines did not indicate bias. The mean weighted prediction errors were not statistically different from 0, while the mean weighted squared prediction errors and the bias‐adjusted mean weighted squared prediction errors were reasonably small. The residuals showed normal distribution and their means were not significantly different from 0. The dispersion of the random effects in marginal plots (Figure 3) was acceptable, without “piling‐up” at either boundary of the defined range or coalescence into a few values with anomalously high probabilities. The posterior pharmacokinetic parameters obtained for ATR+MET included lower K_a_ than that obtained for ATR+ATRL, corresponding to the slower appearance of the metabolites in the circulation, lower K_e_ corresponding to their prolonged presence in the bloodstream, and lower V (Table 2). Several demographic, physiological, and laboratory parameters were tested as candidate covariates, including anti‐HMGCR, the presence of which has been associated with statin myopathy.^26^, ^27^ No strong relationship of any of the tested candidates with random‐effect pharmacokinetic parameters, or clinical status was identified.

Few data have been published on the absorption kinetics of ATR in human adults. The K_a_'s of ATR and ATR+ATRL were fixed at 2.59 h^−1^ and at 3.5 h^−1^, respectively, in two earlier studies.^28^, ^29^ In another one conducted to elaborate a two‐compartment population pharmacokinetic model of ATR and 2OATR in children and adolescents with heterozygous familial hypercholesterolemia, the K_a_ of ATR was estimated as 0.235 h^−1^.^30^ These results are in acceptable agreement with the value we obtained (1.74 h^−1^), but indicate that the K_a_ of ATR may differ considerably in various populations.

The estimated median V/F of ATR+ATRL (376 L) was in agreement with that displayed in the regulatory documents of oral ATR formulations (381 L).^31^ Central compartment volumes of distribution of 3250 L and 2910 L were reported for ATR+ATRL and ATR+2OATR, respectively, following the oral administration of ATR and the construction of two‐compartment models.^28^, ^29^ These discrepancies may be attributed to the differences between the sets of the random‐effect pharmacokinetic parameters estimated by single‐ and two‐compartment pharmacokinetic models.

Based on the equation t_1__/2_ = ln2/K_e_, our estimates of the median systemic half‐lives (t_1/2_) of ATR+ATRL and ATR+MET are 5.1 h and 6.3 h, respectively. These values are lower than that cited in the regulatory documents of ATR formulations (14 h), but are similar to those reported by others.^31^, ^32^, ^33^, ^34^ Lins et al. established mean half‐lives of 11.5 and 10.9 h for ATR after administering high doses (40 mg or 80 mg, respectively).^35^ The proposed reasons for these differences are the evaluation of ATR+ATRL and ATR+MET, the use of a single‐compartment model, and heavily relying on early sample collection.

Monte Carlo simulation and the ROC analysis demonstrated the utility of the developed models in discriminating adherent from partially adherent, as well as partially adherent from nonadherent subjects, with optimal sampling times of 3 h and 1 h postdose, respectively. The predictive value of ATR+ATRL was superior to that of ATR+MET (Figure 4). To evaluate patient adherence, we therefore propose that the first value of interest should be the probability of attaining the measured ATR+ATRL concentration by simulated adherent subjects. The higher this probability value is, the higher the chance of making erroneous judgement by considering the patient adherent. Unless a clear decision cannot be made, the probability value obtained for simulated partially adherent subjects should also be consulted. In any case when the consultation of ATR+ATRL probabilities does not allow a clear‐cut decision, ATR+MET concentrations should be evaluated in the same manner.

Two examples are presented. Subject A1 was classified as partially adherent because a clear decision could not be made based on ATR+ATRL concentrations. In a 12‐hour sample, 79.74% of adherent patients would attain higher concentrations, while almost all partially adherent or nonadherent individuals would attain lower ones. Doubt is cast on the full adherence of this subject, however, on consulting the probabilities associated with ATR+MET concentrations. Here the chance of exceeding the observed concentration in case of full adherence is 95.29%, but only 25.67% for a partially adherent individual (Table 3). The assayed ATR+ATRL and ATR+MET levels together therefore signal partial rather than full adherence. Concerning Subject A5, the probabilities of finding ATR+ATRL and ATR+MET levels in the 16‐hour sample of adherent, partially adherent, and nonadherent subjects higher than those observed are 96.84% and 97.79%, 59.72% and 63.84%, as well as 1.79% and 11.57%, respectively. While full adherence is very unlikely in this case, the small majority of partially adherent patients would attain higher concentrations than those measured. Making a decision therefore requires the evaluation of probabilities associated with nonadherence as well. These probabilities are low, indicating that partial adherence is the best estimate.

We estimate that 38.46% of subjects in the adherence testing set failed to take their last dose. In these subjects, the probabilities of attaining higher ATR+ATRL concentrations in adherent and partially adherent individuals than those measured were 78.40%–100%, and 3.29%–59.72%, respectively, and were 89.94%–100% and 12.81%–64.82%, respectively, regarding ATR+MET concentrations. The probabilities of observing the measured values (0.00%–1.79% and 0.95%–11.57%, respectively) in nonadherent patients were negligible, eliminating the assumption that any of the subjects may have completely abandoned the therapy.

Unelucidated or unattended drug–drug interactions may lead to the erroneous classification of ATR therapy adherence. Most importantly, the immunosuppressants cyclosporine, everolimus, sirolimus, and tacrolimus may increase ATR exposure considerably. The radical reduction of ATR dose and the close monitoring of individual ATR+ATRL and ATR+MET levels are therefore warranted. Gemfibrozil inhibits the metabolism of ATR, while the coadministration of colchicine results in variable changes in drug exposure.^36^

Our study has limitations. ATR is a generic drug available in various per os formulations. The tablet taken by our subjects contained lactose, the abnormal digestion of which may cause changes in the intestinal metabolism and the enterohepatic circulation of ATR. While none reported gastrointestinal adverse effects, subjects were not interrogated for lactose intolerance. The presented approach does not provide unambiguous evidence for the classification of patients regarding their adherence, and the careful clinical validation of the individual classifications must be accomplished before introducing the presented approach into the clinical practice. Finally, no effort was made to differentiate subpopulations by age, gender, dose of ATR, drug taking habits, or pathological conditions.

## 5 CONCLUSIONS

The developed methodology allows the complex, probability‐based evaluation of adherence to ATR therapy by using tools of bioinformatics currently available at no cost. This approach is considerably more realistic and efficient considering the complexity of the pharmacokinetic properties of ATR than making a dichotomous judgement based on trough sampling and a therapeutic range. The best practice of following up patients includes the regular monitoring of ATR+ATRL and ATR+MET concentrations in each individual from the beginning of their treatment.

Improving the quality of laboratory approaches is of pivotal importance, and TDM results should be one of the several components in the complex evaluation of the status of the individual patient. Nevertheless, it should be emphasized that weighing TDM results in making a clinical decision remains the responsibility of the clinician who oversees the therapy.

## CONFLICT OF INTEREST

The authors have no conflict of interest to declare.

## AUTHORS CONTRIBUTIONS

Gellert Balazs Karvaly developed and validated the analytical method of assaying atorvastatin and its metabolites, ran the laboratory records, performed pharmacokinetic modeling, Monte Carlo simulations and statistical evaluation, and drafted the manuscript. István Karádi performed the duties of the principal investigator, coordinated the application for the ethical approval of the study and provided supervision in preparing the manuscript. István Vincze analyzed atorvastatin and its metabolites in serum samples, and wrote the respective methodological section. Michael N. Neely provided the Pmetrics^TM^ software and guidance regarding its use, and revised the manuscript. Eszter Trojnár and Zoltán Prohászka were in charge of the clinical documentation following the de‐identification of subjects, performed the analysis of anti‐HMGCR, and wrote the respective methodological section. Éva Imreh performed clinical chemistry assays, collected and evaluated the analytical data, and contributed the respective methodological description. Barna Vásárhelyi coordinated the laboratory procedures and revised the manuscript. András Zsáry coordinated the recruitment of subjects, the collection and evaluation of the clinical data, and revised the manuscript.

## Supporting information

Supplementary Material

Supplementary Material

Supplementary Material

Supplementary Material

Supplementary Material

Supplementary Material

## Data Availability

Data obtained after the de‐identification of subjects are available from the corresponding author upon a reasonable request.
