# Supplementary material for: A pharmacokinetics‐based approach to the monitoring of patient adherence to atorvastatin therapy
Source: Pharmacol Res Perspect. 2021 Sep 3;9(5):e00856. doi: 10.1002/prp2.856 (PMC8415218; doi:10.1002/prp2.856)
Supplement: Supplementary file 1 — Supplementary Material [file PRP2-9-e00856-s006.docx]

Supporting information 1: detailed demographic and clinical laboratory values of subjects included in (A) the model training set, and (B) the adherence testing set.

| **(A) Model training set** | | | | | | | | | | | | | | |
| --- | --- | --- | --- | --- | --- | --- | --- | --- | --- | --- | --- | --- | --- | --- |
| **ID** | **gender** | **Age (years)** | **Dose (mg)** | **BMI (kg/m^2^)** | **Anti-HMGCR (U/ml)** | **LDL-C (mmol/L)** | **HDL-C (mmol/L)** | **TG (mmol/L)** | **Total cholesterol (mmol/L)** | **Creatine kinase (U/L)** | **Serum creatinine (µmol/L)** | **GPT (U/L)** | **GOT (U/L)** | **LDH (U/L)** |
| M1 | F | 76 | 20 | 28.1 | 3.86 | 3.68 | 1.31 | 1.5 | 5.7 | 215 | 48 | 121 | 101 | 356 |
| M2 | M | 68 | 20 | 26 | 160 | 3.29 | 0.89 | 1.4 | 4.8 | 92 | 60 | 25 | 24 | 140 |
| M3 | M | 74 | 20 | 28.4 | 8.19 | 2.92 | 0.94 | 1.46 | 4.3 | 131 | 153 | 32 | 27 | 239 |
| M4 | M | 63 | 20 | 29.3 | 5.37 | 1.88 | 0.85 | 1.06 | 3 | 134 | 85 | 27 | 30 | 185 |
| M5 | M | 84 | 20 | 25.8 | 1.60 | 2.17 | 0.76 | 0.74 | 3.3 | 102 | 131 | 20 | 25 | 192 |
| M6 | F | 77 | 20 | 33.9 | 5.75 | 3.03 | 0.95 | 1.32 | 4.4 | 142 | 70 | 28 | 26 | 218 |
| M7 | M | 63 | 20 | 30.7 | 3.21 | 2.93 | 1.4 | 24.34 | 12.2 | 30 | 150 | 45 | 29 | 147 |
| M8 | F | 68 | 40 | 28.5 | 7.90 | 1.25 | 0.77 | 0.85 | 2.3 | 73 | 140 | 18 | 23 | 246 |
| M9 | M | 88 | 20 | 24.6 | 8.87 | 2.03 | 0.86 | 1.35 | 3.2 | 31 | 103 | 34 | 32 | 150 |
| M10 | M | 69 | 40 | 44.1 | 4.93 | 1.98 | 0.65 | 6.62 | 2.9 | 178 | 139 | 29 | 33 | 303 |
| M11 | M | 69 | 20 | 28 | 0.00 | 2.52 | 0.92 | 0.43 | 2.2 | 30 | 101 | 16 | 16 | 146 |
| M12 | M | 45 | 40 | 25.6 | 4.13 | 4.5 | 1.23 | 1.65 | 6.7 | 97 | 86 | 22 | 22 | 293 |
| M13 | M | 63 | 20 | 20.2 | 42.1 | 3.1 | 0.86 | 1.44 | 4.6 | 122 | 63 | 26 | 35 | 206 |
| M14 | M | 87 | 20 | 19.1 | 12.3 | 1.76 | 0.87 | 1.08 | 2.9 | 224 | 196 | 17 | 18 | 313 |
| M15 | M | 68 | *20* | 22.9 | 33.0 | 2.9 | 1.06 | 0.76 | 4.3 | 62 | 197 | 27 | 29 | 88 |
| M16 | M | 75 | 20 | 29.1 | 7.99 | 1.38 | 0.83 | 1.35 | 2.6 | 108 | 501 | 14 | 13 | 178 |
| M17 | F | 69 | 40 | 30.1 | 3.66 | 4.15 | 1.53 | 1.29 | 6.5 | 65 | 61 | 15 | 17 | 259 |
| M18 | M | 80 | 40 | 33.3 | 9.31 | 1.47 | 0.58 | 1.43 | 2.6 | 133 | 146 | 19 | 33 | 150 |
| M19 | M | 60 | *20* | 26.5 | 7.41 | 1.99 | 1.29 | 0.63 | 3.4 | 96 | 67 | 61 | 27 | 238 |
| M20 | M | 76 | *20* | 23.4 | 30.8 | 1.24 | 1.09 | 0.72 | 2.7 | 23 | 58 | 12 | 12 | 98 |
| M21 | F | 29 | *20* | 35.3 | 3.26 | 3.86 | 0.99 | 1.17 | 5.6 | 126 | 65 | 12 | 15 | 250 |
| M22 | F | 77 | *20* | 37.3 | 4.19 | 1.86 | 0.81 | 1.08 | 3.1 | 197 | 47 | 23 | 28 | 274 |
| M23 | M | 84 | 40 | 29 | 5.82 | 2.28 | 0.81 | 1.47 | 3.4 | 167 | 163 | 18 | 18 | 164 |
| M24 | F | 75 | 40 | 22.3 | 19.7 | 2.2 | 0.81 | 1.33 | 3.3 | 88 | 58 | 12 | 18 | 173 |
| M25 | F | 71 | 20 | 25.7 | 3.29 | 3.74 | 1.03 | 2.67 | 5.6 | 104 | 85 | 14 | 21 | 166 |
| M26 | F | 84 | 20 | 23.4 | 50.5 | 4.15 | 1.34 | 0.89 | 6.2 | 84 | 102 | 18 | 27 | 200 |
| M27 | M | 66 | 20 | 43.4 | 5.88 | 2.31 | 0.84 | 2.11 | 3.6 | 38 | 74 | 30 | 24 | 141 |
| M28 | M | 76 | 20 | 18.6 | 9.10 | 2.35 | 0.92 | 0.94 | 3.5 | 51 | 73 | 25 | 25 | 172 |
| M29 | M | 46 | 20 | 29 | 15.2 | 2.78 | 1.02 | 0.97 | 4.2 | 49 | 155 | 27 | 26 | 208 |
| M30 | F | 75 | 20 | 17.1 | 7.24 | 2.17 | 0.86 | 1.27 | 1.27 | 78 | 88 | 39 | 56 | 274 |
| M31 | F | 80 | 40 | 34.6 | 11.9 | 2.25 | 1.29 | 1.45 | 3.7 | 22 | 66 | 37 | 43 | 197 |
| M32 | M | 86 | 20 | 34 | 23.5 | 1.32 | 0.81 | 0.72 | 2.4 | 116 | 110 | 21 | 28 | 178 |
| M33 | F | 78 | 20 | 28.8 | 14.5 | 3.8 | 0.78 | 4.08 | 5.4 | 139 | 132 | 15 | 26 | 233 |
| M34 | M | 86 | 20 | 24.4 | 9.19 | 1.66 | 1.04 | 0.73 | 2.9 | 22 | 212 | 14 | 15 | 166 |
| M35 | M | 79 | 20 | 34.9 | 4.45 | 3.36 | 1.72 | 1.12 | 5.7 | 188 | 111 | 16 | 16 | 171 |
| M36 | M | 80 | 40 | 33.3 | 27.4 | 1.6 | 0.76 | 0.49 | 2.3 | 69 | 144 | 13 | 22 | 188 |
| M37 | M | 77 | 20 | 27.5 | 10.9 | 1.94 | 1.02 | 0.92 | 4.2 | 15 | 81 | 26 | 52 | 108 |
| M38 | M | 86 | 40 | 23.8 | 43.2 | 1.46 | 0.82 | 0.95 | 2.7 | 101 | 195 | 18 | 24 | 145 |
| M39 | M | 71 | 40 | 31 | 14.8 | 2.48 | 1.00 | 0.67 | 3.8 | 94 | 105 | 33 | 28 | 228 |

| **(B) Adherence testing set** | | | | | | | | | | | | | | |
| --- | --- | --- | --- | --- | --- | --- | --- | --- | --- | --- | --- | --- | --- | --- |
| **ID** | **gender** | **Age (years)** | **Dose (mg)** | **BMI (kg/m^2^)** | **Anti-HMGCR (U/ml)** | **LDL-C (mmol/L)** | **HDL-C (mmol/L)** | **TG (mmol/L)** | **Total cholesterol (mmol/L)** | **Creatine kinase (U/L)** | **Serum creatinine (µmol/L)** | **GPT (U/L)** | **GOT (U/L)** | **LDH (U/L)** |
| A1 | M | 68 | 40 | 33 | 9.53 | 2.05 | 1.62 | 0.65 | 3.9 | 84 | 81 | 32 | 182 | 182 |
| A2 | F | 76 | 20 | 18 | 4.81 | 2.22 | 1.75 | 1.54 | 4.3 | 126 | 63 | 23 | 203 | 203 |
| A3 | F | 69 | 20 | 17 | 8.67 | 2.97 | 1.77 | 1.09 | 5 | 149 | 68 | 21 | 198 | 198 |
| A4 | F | 66 | 20 | 25 | 0.00 | 2.29 | 1.58 | 1.07 | 4.4 | 83 | 43 | 32 | 242 | 242 |
| A5 | F | 64 | 40 | 19 | 2.03 | 1.75 | 1.2 | 1.73 | 3.2 | 44 | 48 | 39 | 154 | 154 |
| A6 | F | 48 | 20 | 15 | 13.73 | 3.52 | 1.01 | 6.14 | 5.6 | 79 | 44 | 13 | 82 | 82 |
| A7 | M | 72 | 20 | 20 | 0.00 | 2.35 | 0.99 | 1.23 | 3.7 | 65 | 87 | 26 | 191 | 191 |
| A8 | F | 59 | 20 | 27 | 10.78 | 3.43 | 2.01 | 0.93 | 5.8 | 300 | 56 | 36 | 221 | 221 |
| A9 | F | 72 | 20 | 26 | 11.74 | 2.29 | 1.53 | 1.42 | 4.2 | 82 | 64 | 20 | 213 | 213 |
| A10 | M | 71 | 20 | 25 | 46.31 | 2.06 | 1.06 | 1.9 | 3.4 | 96 | 81 | 20 | 185 | 185 |
| A11 | F | 68 | 20 | 16 | 1.27 | 1.82 | 1.56 | 0.7 | 3.4 | 76 | 70 | 17 | 147 | 147 |
| A12 | M | 47 | 40 | 19 | 12.62 | 1.35 | 1 | 0.83 | 2.4 | 127 | 58 | 22 | 168 | 168 |
| A13 | F | 54 | 20 | 27 | 6.71 | 1.93 | 1.32 | 1.27 | 3.5 | 152 | 60 | 19 | 143 | 143 |
| A14 | M | 62 | 40 | 33 | 7.02 | 2.81 | 1.01 | 2.56 | 4.2 | 94 | 73 | 24 | 191 | 191 |
| A15 | M | 77 | 80 | 17 | 63.02 | 1.37 | 1.45 | 0.52 | 2.9 | 81 | 84 | 17 | 190 | 190 |
| A16 | M | 75 | 20 | 13 | 6.54 | 2.37 | 1.62 | 0.86 | 4.2 | 105 | 84 | 17 | 182 | 182 |
| A17 | F | 78 | 10 | 15 | 15.95 | 2.02 | 1.84 | 0.91 | 4.1 | 257 | 73 | 27 | 205 | 205 |
| A18 | M | 78 | 40 | 22 | 3.63 | 1.44 | 1.53 | 0.5 | 3.1 | 49 | 65 | 24 | 152 | 152 |
| A19 | F | 78 | 20 | 16 | 4.89 | 2.12 | 0.77 | 4.43 | 3.5 | 176 | 75 | 32 | 208 | 208 |
| A20 | M | 81 | 20 | 11 | 8.95 | 3.02 | 1.01 | 1.98 | 4.6 | 159 | 115 | 20 | 167 | 167 |
| A21 | F | 81 | 20 | 12 | 5.31 | 2.51 | 0.85 | 3.74 | 4 | 57 | 67 | 14 | 158 | 158 |
| A22 | M | 82 | 20 | 16 | 6.14 | 2.8 | 1.18 | 2.26 | 4.5 | 117 | 82 | 19 | 215 | 215 |
| A23 | F | 73 | 20 | 19 | 10.35 | 2.53 | 1.95 | 0.83 | 4.7 | 316 | 83 | 20 | 214 | 214 |
| A24 | F | 75 | 20 | 27 | 4.9 | 2.37 | 1.6 | 0.96 | 4.3 | 39 | 164 | 29 | 255 | 255 |
| A25 | M | 79 | 80 | 8 | 7.89 | 1.4 | 0.94 | 0.96 | 2.7 | 74 | 84 | 15 | 191 | 191 |
| A26 | F | 81 | 20 | 14 | 13.11 | 2.12 | 1.58 | 1.29 | 4.1 | 82 | 125 | 19 | 208 | 208 |
