# Supplementary material for: A pharmacokinetics‐based approach to the monitoring of patient adherence to atorvastatin therapy
Source: Pharmacol Res Perspect. 2021 Sep 3;9(5):e00856. doi: 10.1002/prp2.856 (PMC8415218; doi:10.1002/prp2.856)
Supplement: Supplementary file 2 — Supplementary Material [file PRP2-9-e00856-s002.docx]

Supporting information 2. Final input population pharmacokinetic model files. (A) ATR+ATRL, (B) ATR+MET.

(A)

#Pri

Ka, 0.01, 9

Ke, 0.1, 0.5

V, 5, 125

FA1, 0.125!

#Cov

IC

#Ini

X(2)=IC*V

#Out

Y(1) = X(2)/V

#F

FA(1)=FA1

#Err

L=0.01

0.0000826, 0.03530, 0, 0!

(B)

#Pri

Ka, 0.001, 4

Ke, 0.01, 0.4

V, 5, 80

FA1, 0.125!

#Cov

IC

#Ini

X(2)=IC*V

#Out

Y(1) = X(2)/V

#F

FA(1)=FA1

#Err

L=0.01

0.0000386, 0.03321, 0, 0!
