# Supplementary material for: A pharmacokinetics‐based approach to the monitoring of patient adherence to atorvastatin therapy
Source: Pharmacol Res Perspect. 2021 Sep 3;9(5):e00856. doi: 10.1002/prp2.856 (PMC8415218; doi:10.1002/prp2.856)
Supplement: Supplementary file 5 — Supplementary Material [file PRP2-9-e00856-s004.docx]

**Supporting information 5.** Pharmacometric details of the constructed population models: modeled ranges of pharmacokinetic parameters and characteristics of the final models. ATR+ATRL model: pharmacokinetic model based on the sums of atorvastatin and atorvastatin lactone concentrations. ATR+MET model: pharmacokinetic model based on the sums of atorvastatin, atorvastatin lactone, 2-hydroxyatorvastatin, 2-hydroxyatorvastatin lactone, 4-hydroxyatorvastatin and 4-hydroxyatorvastatin lactone concentrations.

|  | | ***ATR+ATRL*** | ***ATR+MET*** |
| --- | --- | --- | --- |
| ***Modeled ranges of pharmacokinetic parameters*** | | | |
| Random effects | Absorption rate constant (1/h) | 0.01-9.0 | 0.001-4.0 |
|  | Elimination rate constant (1/h) | 0.1-0.5 | 0.01-0.4 |
|  | Volume of distribution (L) | 5-125 | 5-80 |
| Fixed effect | Bioavailability | 0.125 | 0.125 |
| ***Characteristics of the final population models*** | | | |
| Mean weighted prediction error (p-value of difference from 0) | | -0.027 (0.513) | 0.080 (0.195) |
| Bias-adjusted mean weighted squared prediction error | | 0.677 | 0.768 |
| Mean weighted squared prediction error | | 0.678 | 0.775 |
| Mean of residuals (SD, p-value of difference from 0) | | 0.03 (0.83, 0.713) | 0.08 (0.88, 0.304) |
| p value, Kolmogorov-Smirnov test of the normality of the distribution of residuals | | 0.48 | 0.10 |
| Number of support points | | 21 | 19 |
| Coefficients of the linear correlations between pharmacokinetic parameters | | -0.473 – 0.095 | -0.396 – -0.128 |
| Covariates | | initial concentration | initial concentration |
| Shrinkage of Ka (%) | | 18.3 | 10.6 |
| Shrinkage of Ke (%) | | 21.4 | 5.19 |
| Shrinkage of V/F (%) | | 4.63 | 2.42 |
| -2 x log-likelihood | | -523.7 | -455.3 |
| Akaike information criterion | | -515.4 | -446.9 |
| Bayesian information criterion | | -504.3 | -435.8 |
