# Supplementary material for: A pharmacokinetics‐based approach to the monitoring of patient adherence to atorvastatin therapy
Source: Pharmacol Res Perspect. 2021 Sep 3;9(5):e00856. doi: 10.1002/prp2.856 (PMC8415218; doi:10.1002/prp2.856)
Supplement: Supplementary file 6 — Supplementary Material [file PRP2-9-e00856-s003.docx]

Supporting information 6. Linear correlation P values and correlation coefficients found between the random-effect pharmacokinetic parameters, the demographic values and the measured clinical laboratory parameters.

| Covariate | Random-effect parameter | | | | | |
| --- | --- | --- | --- | --- | --- | --- |
|  | Absorption rate coefficient, K_a_ | | Elimination rate coefficient, K_e_ | | Apparent volume of distribution, V_d_ (L) | |
|  | Linear Correlation P value | Linear correlation coefficient (r) | Linear Correlation P value | Linear correlation coefficient (r) | Linear Correlation P value | Linear correlation coefficient (r) |
| ATR+ATRL | | | | | | |
| Gender | 0.157 | Not applicable | 0.796 | Not applicable | *0.002* | Not applicable |
| Dose | 0.463 | Not applicable | 0.130 | Not applicable | 0.175 | Not applicable |
| Age | 0.951 | 0.010 | 0.971 | 0.006 | 0.064 | 0.300 |
| BMI | 0.145 | 0.237 | 0.680 | 0.068 | 0.778 | 0.047 |
| Serum creatinine | 0.714 | 0.061 | 0.652 | 0.074 | 0.114 | 0.257 |
| Serum total cholesterol | 0.337 | 0.158 | 0.879 | 0.025 | *0.024* | 0.361 |
| Serum creatine kinase | *0.022* | 0.366 | 0.315 | 0.165 | 0.482 | 0.116 |
| Serum LDL-C | 0.405 | 0.137 | 0.328 | 0.161 | *0.032* | 0.343 |
| Serum triglycerides | 0.915 | 0.018 | 0.945 | 0.012 | 0.520 | 0.106 |
| Serum HDL-C | 0.570 | 0.094 | 0.196 | 0.212 | *0.025* | 0.358 |
| Serum LDH | 0.842 | 0.033 | 0.439 | 0.127 | 0.842 | 0.330 |
| Serum GOT | 0.665 | 0.072 | 0.204 | 0.208 | 0.413 | 0.135 |
| Serum GPT | 0.869 | 0.027 | 0.118 | 0.255 | 0.072 | 0.291 |
| Serum anti-HMGCR | 0.587 | 0.090 | 0.593 | 0.088 | 0.557 | 0.097 |
| ATR+MET | | | | | | |
| Gender | 0.533 | Not applicable | 0.867 | Not applicable | 0.378 | Not applicable |
| Dose | 0.743 | Not applicable | *0.026* | Not applicable | 0.268 | Not applicable |
| Age | 0.582 | 0.091 | 0.178 | 0.220 | *0.019* | 0.374 |
| BMI | 0.434 | 0.129 | 0.776 | 0.047 | 0.820 | 0.038 |
| Serum creatinine | 0.249 | 0.189 | 0.520 | 0.106 | 0.114 | 0.257 |
| Serum total cholesterol | 0.259 | 0.185 | 0.614 | 0.083 | *0.021* | 0.370 |
| Serum creatine kinase | 0.124 | 0.250 | 0.388 | 0.142 | 0.313 | 0.166 |
| Serum LDL-C | 0.212 | 0.204 | 0.758 | 0.051 | *0.029* | 0.350 |
| Serum triglycerides | 0.997 | 0.001 | 0.805 | 0.041 | 0.450 | 0.124 |
| Serum HDL-C | 0.445 | 0.126 | 0.054 | 0.311 | 0.111 | 0.259 |
| Serum LDH | 0.948 | 0.011 | 0.752 | 0.052 | 0.975 | 0.005 |
| Serum GOT | 0.345 | 0.155 | 0.196 | 0.212 | 0.599 | 0.087 |
| Serum GPT | 0.866 | 0.028 | 0.149 | 0.236 | 0.131 | 0.246 |
| Serum anti-HMGCR | 0.680 | 0.068 | 0.831 | 0.035 | 0.422 | 0.132 |
